# CXCL5 Modified Nanoparticle Surface Improves CXCR2^+^ Cell Selective Internalization

**DOI:** 10.3390/cells9010056

**Published:** 2019-12-24

**Authors:** Roberta Cagliani, Francesca Gatto, Giulia Cibecchini, Roberto Marotta, Federico Catalano, Paola Sanchez-Moreno, Pier Paolo Pompa, Giuseppe Bardi

**Affiliations:** 1 Nanobiointeractions & Nanodiagnostics, Istituto Italiano di Tecnologia, Via Morego 30, 16163 Genova, Italy; roberta.cagliani@iit.it (R.C.); francesca.gatto@iit.it (F.G.); giulia.cibecchini@iit.it (G.C.); paola.sanchez@iit.it (P.S.-M.); pierpaolo.pompa@iit.it (P.P.P.); 2 Department of Chemistry and Industrial Chemistry, University of Genova, Via Dodecaneso 31, 16146 Genova, Italy; 3 Electron Microscopy Laboratory, Istituto Italiano di Tecnologia, Via Morego 30, 16163 Genova, Italy; roberto.marotta@iit.it (R.M.); federico.catalano@iit.it (F.C.)

**Keywords:** chemokines, chemokine receptors, immune cells, nanoparticles, surface chemistry

## Abstract

Driving nanomaterials to specific cell populations is still a major challenge for different biomedical applications. Several strategies to improve cell binding and uptake have been tried thus far by intrinsic material modifications or decoration with active molecules onto their surface. In the present work, we covalently bound the chemokine CXCL5 on fluorescently labeled amino-functionalized SiO_2_ nanoparticles to precisely targeting CXCR2^+^ immune cells. We synthesized and precisely characterized the physicochemical features of the modified particles. The presence of CXCL5 on the surface was detected by z-potential variation and CXCL5-specific electron microscopy immunogold labeling. CXCL5-amino SiO_2_ nanoparticle cell binding and internalization performances were analyzed in CXCR2^+^ THP-1 cells by flow cytometry and confocal microscopy. We showed improved internalization of the chemokine modified particles in the absence or the presence of serum. This internalization was reduced by cell pre-treatment with free CXCL5. Furthermore, we demonstrated CXCR2^+^ cell preferential targeting by comparing particle uptake in THP-1 vs. low-CXCR2 expressing HeLa cells. Our results provide the proof of principle that chemokine decorated nanomaterials enhance uptake and allow precise cell subset localization. The possibility to aim at selective chemokine receptor-expressing cells can be beneficial for the diverse pathological conditions involving immune reactions.

## 1. Introduction

The search for nanomaterials (NMs) with the ability to travel to specific biological tissues is constantly increasing. Several nanoparticles (NP) engineering methods [1] as well as specific corona formation [2] have been tested thus far. However, many physical, chemical, or biological barriers limit their successful application. In vertebrates, the major physiological protection towards the invasion of external bodies is provided by the immune system (IS), the first line of which involves the so called “innate” IS. This is formed by a huge number of molecules with binding and enzymatic activity that opsonize and modify the alien threat, allowing its quick removal by phagocytes. As drawback for medicine, phagocytosis often stops the route of many drug delivery systems to reach their final destination, pushing the researchers to find ways to escape this trapping machine. A safe delivery is even more intricate if the targets are other immune cells, which share many tissue environments with phagocytes.

In the last decades, several strategies have been developed to improve the cellular uptake and the fate of nanoparticles [3,4]. Among them, the surface decoration with biological compounds has expanded the biomedical applications of insoluble and inorganic materials [5]. For example, the immobilization of peptides on the surface of gold NPs can result in their improved stability in colloidal suspensions as well the exploitation of the ligand–receptor mechanism to increase cellular internalization [6,7]. Numerous nanomaterials, however, show the intrinsic property to adsorb specific proteins present in biological fluids (e.g., blood) that can be used as targeting molecules to drive cell localization [8]. Although the formation of a selective protein corona is attractive, it cannot exclude some concerns regarding the non-advantageous peptide orientation or the presence of conformational changes due to the chemical and the physical interactions with the particle surface [9]. Theoretically, the presentation of an entire protein covalently bound on the NP surface could guarantee its correct folding and exposition to the receptor docking site, allowing the NP to exploit the following receptor mediated pathway [10].

We approached the design of a targeting nano-system considering its ability to localize to specific immune cell subsets. Among the many proteins that could allow our purpose, we chose chemokines (*chemo*-tactic cyto-*kines*) [11,12]. These are small proteins consisting of 70–100 amino acids secreted by almost all the cell types. They perform many tasks, including communication among different cells, regulation of hematopoiesis, tissue rearranging, and guide leukocyte migration during inflammation and homing [13,14]. Our choice was based on the stability of these polypeptides constantly released in biological fluids and traveling to the different tissues. Chemokines are characterized by the presence of cysteine residues that define four families depending on the number of cysteines and the presence or not of amino acids in between them (i.e., CXC, CC, C, and CX3C) [15]. They also show a highly conserved tertiary structural fold consisting of three-stranded β-sheet and an α-helix, though their quaternary structures may significantly vary with the sub-families [16].

The biological effects of chemokines are mediated by a plethora of Gα_i_ protein-coupled seven transmembrane spanning receptors evolutionarily classified with a nomenclature that recall their cognate chemokine (i.e., CCR, CXCR, CX3CR, XCR) and a distinguishing number [17]. It is worth mentioning that new and atypical receptors are constantly discovered, and the nomenclature is regularly updated [18]. Remarkably, immune cell subsets are often defined by the chemokine receptors expressed on their surface. The receptors’ patterns can also change during leukocyte differentiation to regulate their physiological and pathological activity (i.e., CCR7 in lymphocytes and dendritic cells) [19,20].

Based on our experience in chemokine receptor binding, signaling, and cell internalization [21,22,23] as well as in SiO_2_-NPs’ interaction with cells [24,25,26], we synthesized and functionalized positively customized SiO_2_-NPs with covalently bound CXCL5. This chemokine, once called epithelial-derived neutrophil-activating peptide 78 (ENA-78), is an important mediator of inflammation, playing crucial roles in the recruitment of neutrophils and other immune cells [27]. It is released by several type of tissues, including endothelial cells and human skin cells after UV radiation [28]. It is also involved in cancer proliferation in different tissues [29,30,31]. Its clinical value for cancer prognosis is also recently becoming very significant [32,33].

In the present work, we fully characterized the physicochemical features of the NPs and the presence of the chemokine onto the NP surface. We hypothesized that the chemokine–receptor binding could improve NP localization to CXCR2 expressing leukocytes. To investigate the targeting potential of our CXCL5-NPs, we evaluated their internalization in CXCR2^+^ THP-1 cells in different in vitro conditions. Furthermore, we analyzed their performances in low expressing cells by flow cytometry and confocal microscopy.

Our results strongly suggest that chemokines can be employed to functionalize NPs for precise immune cell targeting.

## 2. Materials and Methods

### 2.1. SiO_2_ Nanoparticles Preparation, Functionalization, and Characterization

#### 2.1.1. FITC-SiO_2_ Nanoparticle Synthesis

Fluorescent SiO_2_ NPs were synthesized as previously reported [34]. Briefly, *N*-1-(3-trimethoxysilylpropyl)-*N*′-fluoresceyl thiourea (FITC-APTMS) conjugate solution was prepared by dissolving 2 mg fluorescein isothiocyanate (FITC) (Sigma-Aldrich, St. Louis, MO, USA) in 1 mL anhydrous ethanol (Carlo Erba Reagents S.r.l., Milano, Italy) and immediately mixed with 10 µL of (3-Aminopropyl)trimethoxysilane (APTMS) (Sigma-Aldrich, St. Louis, MO, USA) under shaking at room temperature for 4 h in the dark. Under nitrogen atmosphere, 25 mL of ethanol was added to 1 mL of aqueous ammonia (28%) (Sigma-Aldrich, St. Louis, MO, USA) and stirred. Then, 950 µL Tetraethyl orthosilicate (TEOS) (Sigma-Aldrich, St. Louis, MO, USA) was mixed with the conjugate solution, and the reaction was stirred at 600 rpm at room temperature for a further 20 h in darkness. The resulting particles were washed three times by centrifugation and resuspension in EtOH using bath sonication and ultimately resuspended in EtOH to reach a final particle concentration of 10 mg/mL.

#### 2.1.2. Nanoparticle Surface Amination

FITC-SiO_2_ NPs were centrifugated and dispersed in water. Then, 0.5% of acetic acid (Sigma-Aldrich, St. Louis, MO, USA) and 1% of APTMS were added. The reaction was taken under stirrer for 1 h at room temperature. NPs were washed twice and dispersed in water.

#### 2.1.3. Covalent Coupling with CXCL5

NH_2_-SiO_2_ NPs (500 µg/mL) were mixed with CXCL5 (4 µM) (Peprotech, Rocky Hill, NJ, USA) in water, then 4-(4,6-Dimethoxy-1,3,5-triazin-2-yl)-4-methylmorpholinium chloride (DMTMM) (Fluorochem Ltd., Hadfield, UK) 10 µM was added, and the reaction was stirred at room temperature for 4 h. The solution was then centrifugated twice (1100 rpm for 1.5 min) using 100 kDa Amicon tubes (Merck Millipore, Burlington, MA, USA), and the NPs were dispersed in water at the same initial concentration.

#### 2.1.4. Transmission Electron Microscopy

NPs were characterized by TEM using a JEOL JEM-1400 Plus (Jeol, Akishima-shi, Japan), with LaB6 thermionic source with an acceleration voltage of 120 kV. Images were acquired using a Gatan CCD camera Orius 830 (2048 × 2048 active pixels). The particle size distribution was obtained by manual measurement of the diameter of at least 500 NPs using ImageJ software.

#### 2.1.5. Nanoparticle Hydrodynamic Diameter and Surface Charge Measurements

Dynamic light scattering (DLS) measurements of NPs were performed in water at 25 °C using the Zetasizer Nano ZS90 (Malvern, UK). The refractive index (Ri) and the adsorption index (Rabs) of silica were 1.47 and 0.000, respectively, according to the standard operating procedure (SOP) [35,36] Numerical data were kindly provided by F. Lemarchand. **ζ**- potential measurements were made using the Smoluchowski model.

#### 2.1.6. Nanoparticle Immunolabeling

Plasma-cleaned formvar carbon film-coated 300 mesh copper grids (Electron Microscopy Sciences, Hatfield, PA, USA) were incubated 2 min with 5 μL drops containing CXCL5 SiO_2_ NPs. Dilutions were optimized for each SiO_2_ NPs concentration. After two washing steps with 50 μL drops of washing buffer [0.1% bovine serum albumin (BSA) (Miltenyi Biotec, Bergish, Germany) in phosphate buffered saline (PBS) (Sigma-Aldrich, St. Louis, MO, USA)], samples were incubated with 50 μL drops containing buffer A (1% BSA in PBS) for negative control or Rabbit Anti-Human CXCL5 primary antibody (Peprotech, Rocky Hill, NJ, USA) (8 μg/mL in buffer A) in a wet chamber for 3 h at room temperature. After five washes with washing buffer, SiO_2_ NPs were incubated with 20 μL drops of 1:40 dilutions of the secondary antibody {goat anti-rabbit IgG-gold conjugate (H&L) [EM-grade, 10-nm particle size (Electron Microscopy Sciences, Hatfield, PA, USA)]} for 30 min in washing buffer. The system was then washed five times with five drops of washing buffer, followed by five washes with water drops.

### 2.2. Cell Culture

THP-1 cells (ATCC Manassas, VA, USA) were cultured in RPMI-1640 (Thermo Fisher Scientific, Waltham, MA, USA) supplemented with 10% fetal bovine serum (FBS) or 5% human serum (HS) (Thermo Fisher Scientific, Waltham, MA, USA), 1% Penicillin-Streptomycin (Sigma-Aldrich, Saint Louis, MO, USA), and 0.05 mM 2-mercaptoethanol (Thermo Fisher Scientific, Waltham, MA, USA). HeLa cells (ATCC Manassas, VA, USA) were cultured in Dulbecco’s Modified Eagle Medium (DMEM) (Thermo Fisher Scientific, Waltham, MA, USA) supplemented with 10% FBS and 1% Penicillin-Streptomycin. Both the cell lines were grown at 37 °C in a 5% CO_2_ humidified atmosphere.

### 2.3. Cellular Uptake

THP-1 and HeLa cells (2 × 10^5^) were incubated with 50 µg/mL fluorescent NPs for 45 min at 37 °C. After the incubation time, the samples were washed three times at 4 °C. Resulting cell fluorescence was evaluated by flow cytometry. A previous treatment with 1 μM CXCL5 for 45 min was applied before NP incubation where described.

### 2.4. CXCR2 Expression

CXCR2 expression was analyzed incubating THP-1 and HeLa cells (5 × 10^5^ cell/mL) in serum free medium containing 0.5% BSA (Miltenyi Biotec, Bergish, Germany) with APC-conjugated mouse anti-human CXCR2 antibody (Miltenyi Biotec, Bergish, Germany) at the manufacturer’s recommended concentration for 15 min on ice in the dark.

### 2.5. Statistical Analysis

Data were expressed as mean ± standard error of the mean (SEM). For statistical analysis, GraphPad Prism 8 software was used (San Diego, California, CA, USA). *p*-values were calculated using two-tailed *t*-test.

## 3. Results

### 3.1. NP Synthesis and Characterization

#### 3.1.1. SiO_2_ Synthesis, Surface Amination, and CXCL5 Functionalization

**Figure 1:**
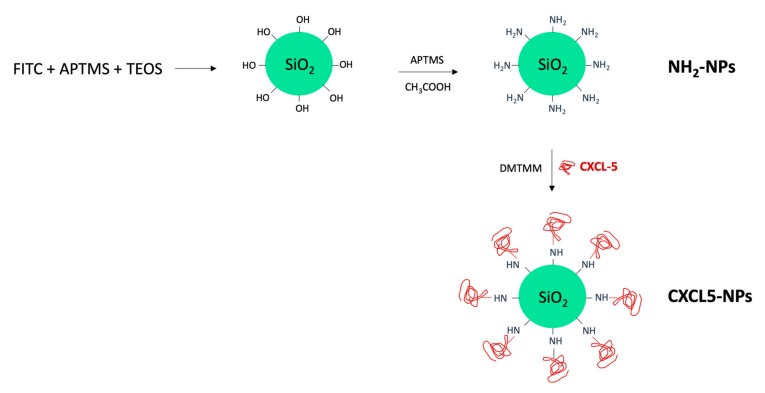
Schematic representation of SiO_2_ nanoparticles (NPs) synthesis.

Fluorescently labeled SiO_2_ NPs were synthesized by co-condensation of *N*-1-(3-trimethoxysilylpropyl)-*N*′-fluoresceyl thiourea (FITC-APTMS) and Tetraethyl orthosilicate (TEOS), as schematically represented in Figure 1. A following reaction step in the presence of APTMS in acetic acid allowed the NP surface amination required for the correct orientation of the chemokine during the subsequent linking reaction. Human CXCL5 was finally conjugated to the NH_2_-SiO_2_ NP surface using the coupling reagent 4-(4,6-Dimethoxy-1,3,5-triazin-2-yl)-4-methylmorpholinium chloride (DMTMM) (Figure 1).

#### 3.1.2. NPs Characterization

**Figure 2:**
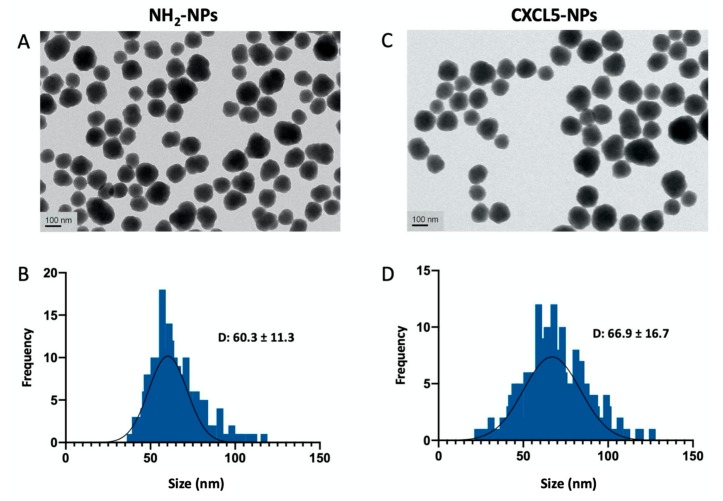
NPs characterization. Transmission electron microscopy (TEM) of NH_2_-NPs (**A**) and CXCL-NPs (**C**). (**B**,**D**) Histograms of size distribution in nm. D value (diameter) reports the average ± standard deviation (SD) in nm. The particle diameter size distribution was obtained by the measurement of at least 500 NPs.

As shown in Figure 2 and Figure S1, transmission electron microscopy (TEM) analysis of the NPs was performed to examine the morphology of the particles that appeared spheroidal with size distribution showing an average diameter of 60.3 ± 11.3 nm for the NH_2_-SiO_2_ NPs (from now on reported as NH_2_-NPs) and 66.9 ± 16.7 for the CXCL5-NH_2_-SiO_2_ NPs (from now on reported as CXCL5-NPs).

The analysis of the hydrodynamic diameter of the CXCL5-NPs in water suspension evaluated by dynamic light scattering (DLS) reports an increase of roughly 20 nm, as expected (Table 1). The surface charge, on the contrary, decreased on average from 29.8 to 16.8 mV according to the chemokine functionalization.

**Figure 3:**
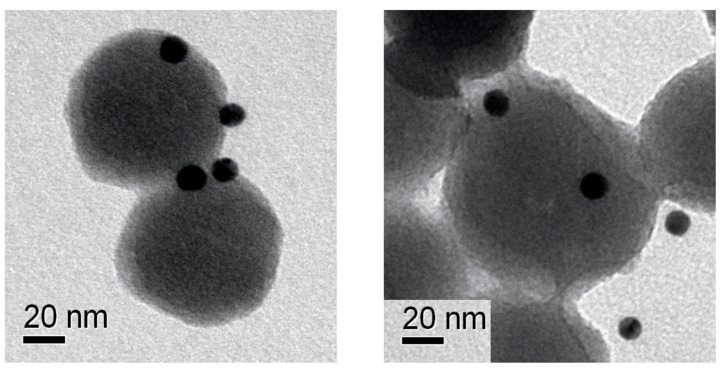
NPs immunolabeling. TEM images showing the epitope mapping of covalently bound CXCL5 on NPs surface by 10 nm immune-gold NPs (dark spots).

To further prove the presence of the chemokine onto the NP surface, we performed epitope mapping of CXCL5 by nanogold conjugated with specific anti-CXCL5 antibodies. Figure 3 shows electron microscopy images of immunogold-labeled NPs. Very electron dense (dark) spots indicate the nanogold labeling of the chemokine, demonstrating its localization onto the NP surface. As shown in Figure 3/right, some spots in the side view apparently seemed detached from the NPs, although the distance was compatible with the antibody size.

### 3.2. Enhanced Cellular Uptake of CXCL5-NPs in CXCR2^+^ Cells

**Figure 4:**
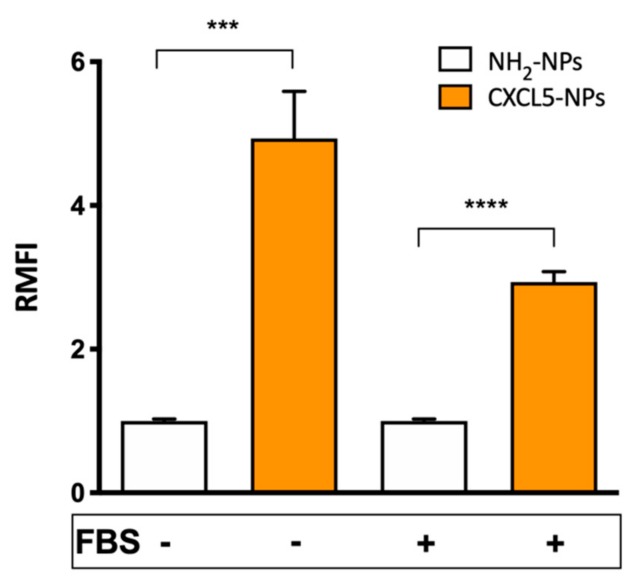
Cellular uptake of NPs. Flow cytometry evaluation of 50 µg/mL NPs internalization in THP-1 cells. The bars represent the relative median fluorescence intensity (RMFI) of at least three independent experiments ± standard error of the mean (SEM). **** *p* < 0.0001 *** *p* < 0.0005.

CXCL5-NP specific targeting properties were studied in CXCR2 expressing myeloid pro-monocytic THP-1 cells [37,38]. We compared the cellular uptake of the chemokine modified particles with the internalization of non-functionalized positively charged NPs used as control, either in the absence or in the presence of serum during the incubation time. Flow cytometry results showed > 4 times increased uptake of the CXCL5-NPs vs. NH_2_-NPs following incubation without serum (Figure 4). Internalization of CXCL5-NPs in THP-1 cells in complete medium was also noticeably enhanced in respect to control particles, although it was less pronounced than in the serum-free condition. This reduction was likely due to the serum diverse protein corona reducing NPs interaction with cells. The intracellular localization of the particles was also confirmed by 3D confocal microscopy highlighting the presence of CXCL5-NPs inside the cells, many of them not colocalizing with lysosomes, as was predictable by the short time incubation (Figure S2).

### 3.3. Receptor Mediated Internalization

**Figure 5:**
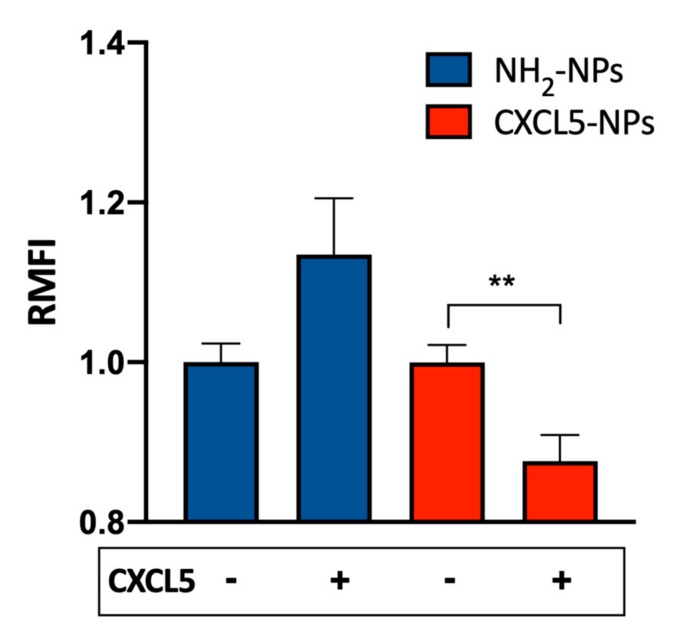
CXCL5 displacement by free CXCL5. Internalization of 50 µg/mL NPs in THP-1 cells pretreated or not with 1 μM CXCL5. The bars represent the relative median fluorescence intensity (RMFI) of at least three independent experiments ± standard error of the mean (SEM). ** *p* < 0.001.

To evaluate the contribution of the CXCR2 to the enhanced cellular uptake of CXCL5-NPs, we pre-treated THP-1 cells with 1 μM CXCL5 and subsequently incubated the cells with both types of NPs. As shown in Figure 5, CXCL5-NP internalization was reduced, whereas THP-1 uptake of NH_2_-NPs revealed a statistically non-significant increase in the average. We hypothesized that the presence of free chemokine in the medium could have created a CXCL5 corona facilitating the entrance of NH_2_-NPs (Cagliani et al. unpublished data). On the contrary, the moderate but statistically significant reduction of CXCL5-NPs internalization may have been due to the agonist-mediated partial internalization of CXCR2 in THP-1 cells. As demonstrated in Figure S3, the administration of saturating concentrations (up to 1 μM) of CXCL5 internalized less than 50% of the receptor expressed on the surface. These data clearly show the different behaviors of CXCL5-NPs and NH_2_-NPs in the presence of receptor occupancy and desensitization by its cognate chemokine.

### 3.4. Selective Targeting of CXCR2^+^ Cells

**Figure 6:**
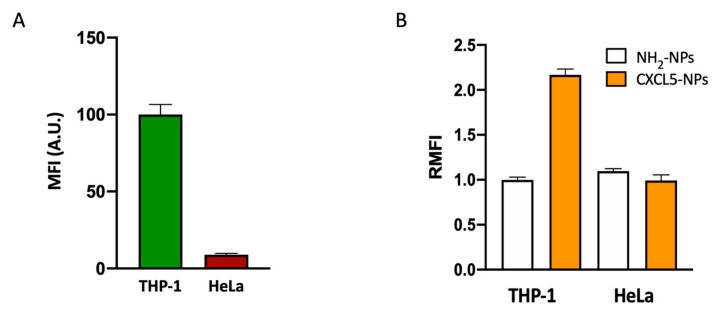
CXCR2^+^ cell preferential targeting of CXCL5-NPs. CXCR2 expression (**A**) and NPs uptake in THP-1 and HeLa cells in serum free conditions (**B**) evaluated by flow cytometry. The bars represent the median fluorescence intensity (MFI) and the relative median fluorescence intensity (RMFI) of at least three independent experiments ± standard error of the mean (SEM).

In order to study the targeting selectivity of CXCR2^+^ cells, we compared the uptake of NPs in CXCR2^high^ THP-1 cells vs. CXCR2^low^ HeLa cells (Figure 6). The different expression of CXCR2 in these two cell lines was confirmed by flow cytometry (Figure 6A). Incubation of these two cell lines with functionalized and non-functionalized NPs displayed opposite results. In CXCR2^low^ HeLa cells, we could not detect increased CXCL5-NPs internalization with respect to the control NPs (Figure 6B, Figure S4).

The result shown by flow cytometry and confocal microscopy demonstrated the preferential targeting of CXCR2^+^ cell by CXCL5 decorated NPs.

## 4. Discussion

We focused on NP surface functionalization with stable biomolecules that usually interact with all the different immune cells in a selective manner. Based on the role that chemokines have in the physiological and the pathological regulation of immune cells as well as their structural stability in biological media, we decided to modify a prototype NP with these 10 kDa proteins and explore their cell targeting performances. To the best of our knowledge, this is the first work showing detailed synthesis and deep characterization of chemokine functionalized SiO_2_NPs and their specific interaction with immune cells bearing specific chemokine receptors.

The amination of the SiO_2_ NPs was required to permit the functional orientation of the chemokine by increasing the chances of its COOH terminus to link the NP positive surface, keeping the chemokine NH_2_-terminus free. Since the latter was the CXCR2 binding site, this allowed the immobilized CXCL5 to trigger the receptor signaling followed by its cellular internalization. In fact, CXCL5-NPs showed fast and selective targeting of cells expressing the CXCL5-cognate receptor CXCR2. As previously demonstrated [24,25,26], pristine or positively charged SiO_2_-NPs virtually enter every type of cell, giving them enough contact time and appropriate conditions. Nevertheless, the fast chemokine receptor binding can represent the targeting plus of our NPs to avoid their removal or unspecific internalization once released. The CXCL5-NP increased internalization in the presence of serum, which demonstrates the advantage of covalent NP surface functionalization. The expected serum-protein corona formation in the medium seemed to only partially affect the enhanced cellular uptake of our particles. Albeit these results were obtained in vitro, this is encouraging information pushing us to study CXCL5-NP behavior in vivo.

As with CXCL5, all soluble chemokines can be chosen as targeting moieties of NP surfaces. Intriguingly, this modification opens the way to several applications for medical disorders requiring NP–leukocyte interaction. Since precise patterns of receptors are expressed by different immune cells acting in specific tissues and pathological conditions, the choice of the appropriate chemokine could allow the specific release of therapeutics by “ad hoc” modified delivery systems.

The competitive binding between CXCL5-NPs and the free chemokine also reveals a conceivable novel NP-based antagonist role. It is worth mentioning, however, that CXCL5-NP internalization was only partially reduced, probably because almost 50% of CXCR2 expressed on THP1 was not internalized, even with high concentration of free CXCL5. It can be speculated that this event may have been due to saturation of intracellular internalization mechanisms, for example, β-arrestins pathway desensitization [39] or aberrant expression of this receptor in THP-1 cell line. The binding affinity and the signal transduction evoked by CXCL5-NPs binding to CXCR2 are not yet known and are currently being investigated by our group. It will be crucial to understand whether the presence of these particles can limit or impair the biological events induced by the chemokines, such as chemotaxis, intracellular calcium release, or respiratory burst. Although SiO_2_ NPs have been used as model particles for a proof of principle, it will be reasonable to employ biodegradable NPs for future applications for therapeutic aims.

## 5. Conclusions

In this work, we described a novel functionalization of potential nano-delivery systems using chemokines. We synthesized and characterized SiO_2_-NPs by modifying their chemical surface on purpose. We demonstrated that CXCL5 covalently bound onto the surface improved NP internalization into CXCR2^+^ cells. Moreover, the fast chemokine-receptor binding favored the localization to specific receptor-positive cells. Our results prove the principle that chemokines can be used as specific functionalization moieties for delivery systems targeting immune cells. 

## Figures and Tables

**Table 1:** Hydrodynamic diameter, polydispersity index (PDI), and surface charge of the different NPs.

	Hydrodynamic Diameter (nm)	PDI	Zeta Potential (mV)
**SiO_2_ NPs**	94.5 nm ± 22.6	0.1	−50.0 mV ± 7.5
**NH_2_-NPs**	99.4 nm ± 26.8	0.2	29.8 mV ± 5.4
**CXCL5-NPs**	121 nm ± 41.6	0.2	16.8 mV ± 3.2

## Supplementary Materials

The following are available online at https://www.mdpi.com/2073-4409/9/1/56/s1, Figure S1: As-synthesized FITC-SiO_2_ NPs characterization, Figure S2: Confocal microscopy of THP-1 cells treated with 50 µg/mL SiO_2_ NPs for 45 min, Figure S3: Flow cytometry analysis of THP-1, Figure S4: Confocal microscopy of THP-1 and HeLa cells treated with 50 µg/mL of NPs for 45 min.
