# Supplementary material for: CXCL5 Modified Nanoparticle Surface Improves CXCR2+ Cell Selective Internalization
Source: Cells. 2019 Dec 24;9(1):56. doi: 10.3390/cells9010056 (PMC7016632; doi:10.3390/cells9010056)
Supplement: Supplementary file 1 [file cells-09-00056-s001.pdf]

Appendix A

Supporting Information

SiO_2 nanoparticle synthesis and characterization

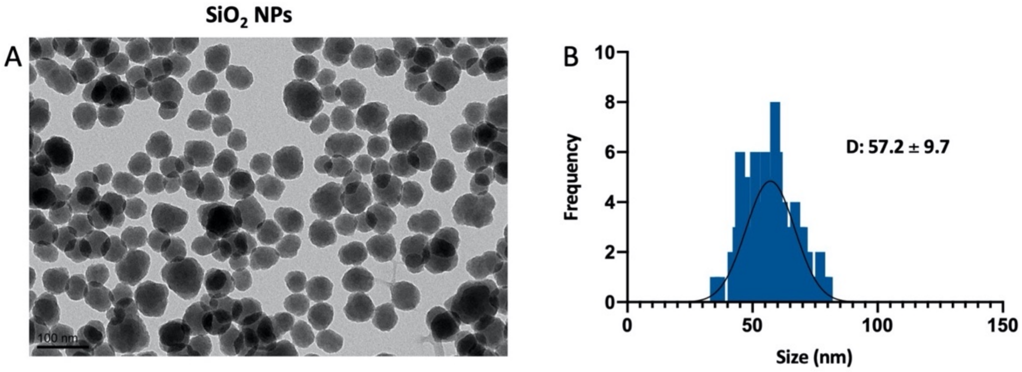

C

	Hydrodynamic diameter (nm)	PDI	Zeta potential
SiO_2 NPs	94.5	0.1	-50.0 mV

Figure S1. As-synthesized FITC-SiO_2 NPs characterization. (**A**) TEM. (**B**) Size distribution measured by TEM and average diameter ± SD value. (**C**) Characterization of NPs in water by dynamic light scattering (DLS).

CXCL5 internalization in THP-1 cells: confocal microscopy

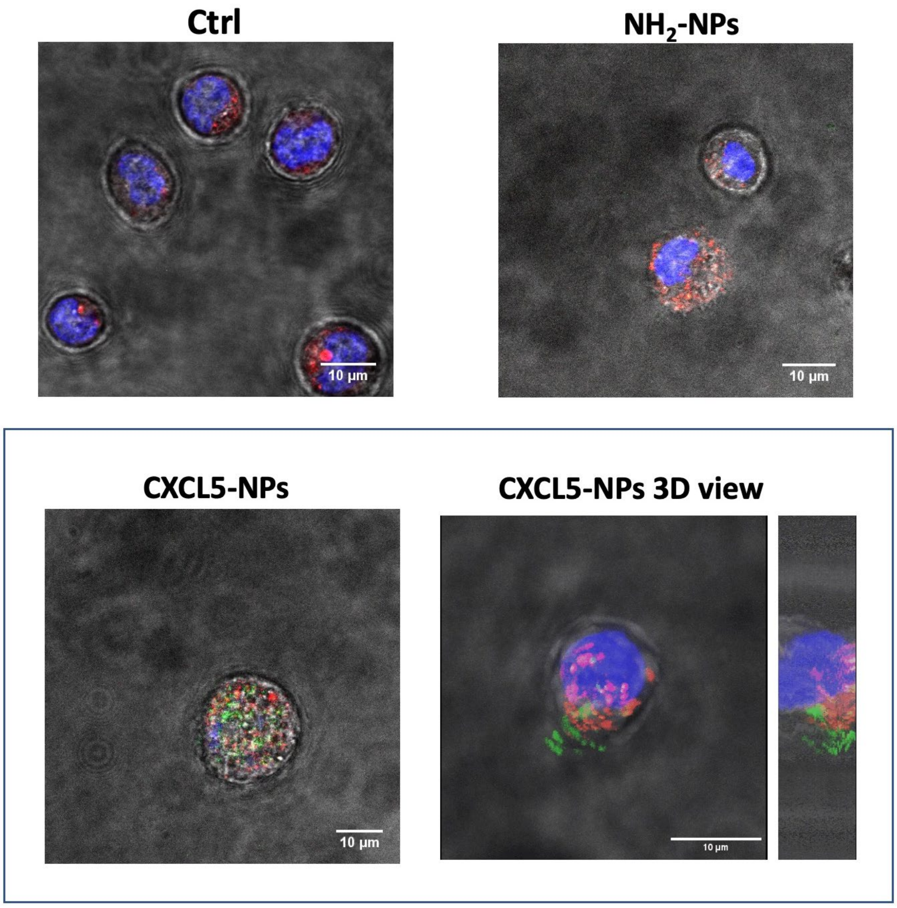

Figure S2. Confocal microscopy of THP-1 cells treated with 50 µg/mL SiO_2 NPs for 45 min. Blue: nuclei; red: lysosomes; green: NPs. Lower right panel represents the maximum projection of z-stack along z-axis,APC and the lateral box represents the maximum projection of z-stack along y–z axis.

CXCR2 expression and internalization

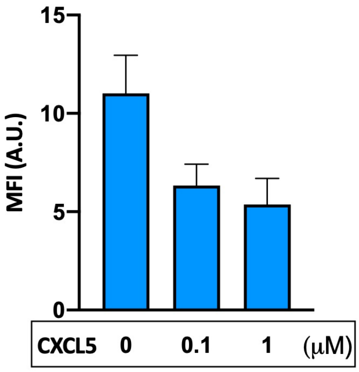

Figure S3. Flow cytometry analysis of THP-1. Bars represent the median fluorescence intensity (MFI) of APC-labeled anti-CXCR2 in the absence or presence of CXCL5.

Confocal microscopy images of nanoparticle treated THP-1 and HeLa cells

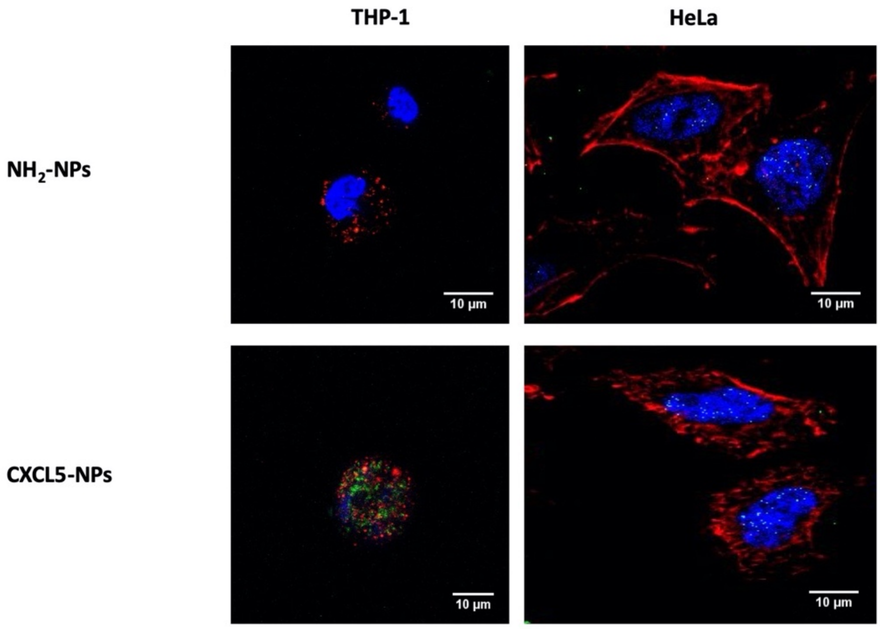

Figure S4. Confocal microscopy of THP-1 and HeLa cells treated with 50 μg/mL of NPs for 45 min. Blue: nuclei; red: lysosomes (THP-1); cytoskeleton (HeLa); green: NPs.

Appendix B

Materials and Methods for Supporting Information

Receptor Staining and Flow Cytometry Analysis

CXCR2 internalization. THP-1 cells were serum starved in RPMI/0.5% BSA for 1 h and then treated with CXCL5 at different concentrations for 1.5 h. After the treatments, the cells were washed and incubated with APC-conjugated mouse anti-human CXCR2 antibody (Miltenyi Biotec, Bergish, Germany) for 15 min in the dark. CXCR2 expression was analyzed by flow cytometry with MACSQuant Analyzer (Miltenyi Biotec, Bergish, Germany) using MACSQuantify software. Living cells were gated based on light forward scattering (FSC) and side scattering (SSC).

Confocal Microscopy

THP-1 and HeLa cells were seeded on a 4 well NUNC Lab-Tek Chambered Coverglass (Thermo Fisher Scientific, Waltham, MA, USA) and incubated in serum free medium with the proper SiO2 NPs at the concentration of 50 μg/mL for 45 min, then washed twice. THP-1 cells were incubated with LysoTracker (Thermo Fisher Scientific, Waltham, MA, USA) and Hoechst 33342 (Thermo Fisher Scientific, Waltham, MA, USA) at a concentration of 5 μg/mL for 15 min at 37 °C. After the staining, the cells were washed twice, and Live Cell Imaging Medium (Thermo Fisher Scientific, Waltham, MA, USA) was added for living cell analysis. HeLa cells were fixed with 4% paraformaldehyde (Sigma-Aldrich, St. Louis, Missouri, USA) for 15 min at room temperature, permeabilized with 0.1% Triton (Sigma-Aldrich, St. Louis, Missouri, USA) for 30 min, and blocked with PBS/1% BSA for 30 min. The cells were then incubated with 0.1 nM Rhodamine Phalloidin (Thermo Fisher Scientific, Waltham, MA, USA) for actin microfilaments staining and 5 μg/mL Hoechst 33342 (Thermo Fisher Scientific, Waltham, MA, USA) for 2 h at room temperature in the dark. Confocal microscopy images were acquired by a Leica SP5 Inverted confocal microscope with a 63 × oil immersion objectives, 405, 495 and 561 nm excitation laser wavelengths and a resolution 1024 × 1024 pixels.
